# Molecular Characterisation of Flavanone *O*-methylation in *Eucalyptus*

**DOI:** 10.3390/ijms23063190

**Published:** 2022-03-16

**Authors:** Krishna Somaletha Chandran, John Humphries, Jason Q.D. Goodger, Ian E. Woodrow

**Affiliations:** 1 School of BioSciences, The University of Melbourne, Parkville, VIC 3010, Australia; ksomaletha@student.unimelb.edu.au (K.S.C.); john.humphries@unimelb.edu.au (J.H.); 2 School of Ecosystem and Forest Sciences, The University of Melbourne, Parkville, VIC 3010, Australia; i.woodrow@unimelb.edu.au

**Keywords:** biosynthesis, flavonoid, gland, methyltransferase, OMT, secondary metabolite, unsubstituted B-ring flavanone

## Abstract

Flavonoids are ubiquitous polyphenolic compounds in plants, long recognised for their health-promoting properties in humans. Methylated flavonoids have received increasing attention due to the potential of methylation to enhance medicinal efficacy. Recently, *Eucalyptus* species with high levels of the *O*-methylated flavanone pinostrobin have been identified. Pinostrobin has potential commercial value due to its numerous pharmacological and functional food benefits. Little is known about the identity or mode of action of the enzymes involved in methylating flavanones. This study aimed to identify and characterise the methyltransferase(s) involved in the regiospecific methylation of pinostrobin in *Eucalyptus* and thereby add to our limited understanding of flavanone biosynthesis in plants. RNA-seq analysis of leaf tips enabled the isolation of a gene encoding a flavanone 7-*O*-methyltransferase (*EnOMT1*) in *Eucalyptus*. Biochemical characterisation of its in vitro activity revealed a range of substrates upon which EnOMT1 acts in a regiospecific manner. Comparison to a homologous sequence from a *Eucalyptus* species lacking *O*-methylated flavonoids identified critical catalytic amino acid residues within *EnOMT1* responsible for its activity. This detailed molecular characterisation identified a methyltransferase responsible for chemical ornamentation of the core flavanone structure of pinocembrin and helps shed light on the mechanism of flavanone biosynthesis in *Eucalyptus*.

## 1. Introduction

Flavonoids are ubiquitous polyphenolic compounds in plants long recognised for their health-promoting properties in humans [1,2]. Methylated flavonoids have received increasing attention due to the potential of methylation to alter biochemical properties and consequently enhance medicinal efficacy. The process of methylation in plants is catalysed by methyltransferases that transfer methyl groups to their substrate. This is an important modification of flavonoids that can improve intestinal absorption or increase metabolic stability, thereby enabling extension of the promising medicinal effects from in vitro to in vivo [3,4].

**Figure 1:**
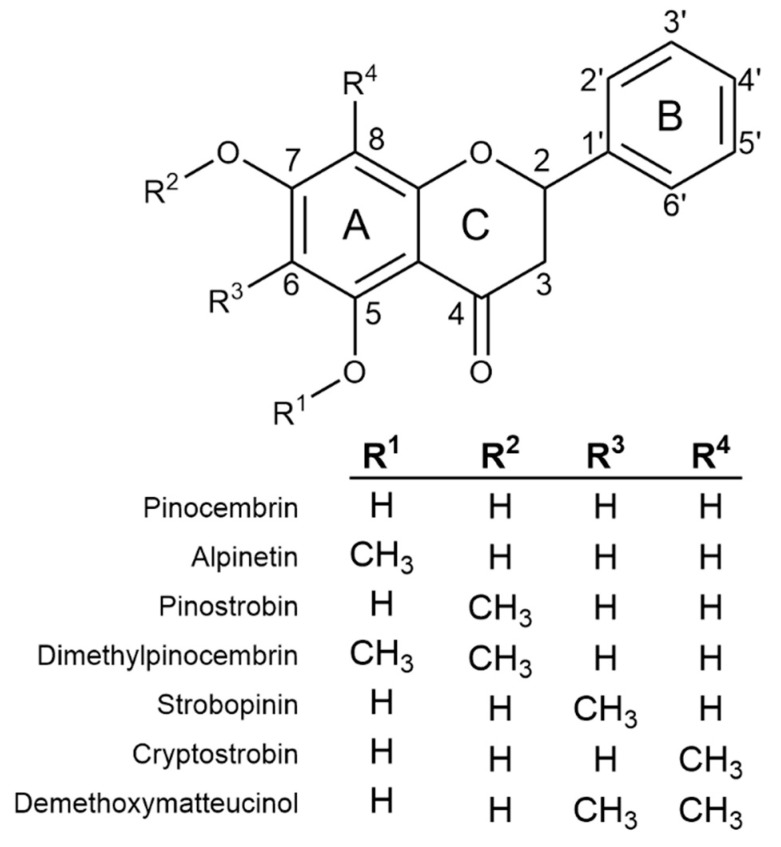
The chemical structure of the core flavanone pinocembrin, together with all possible monomethylated forms and the two most abundant dimethylated flavanones identified from *Eucalyptus*.

Previous screens of plants in genus *Eucalyptus* have shown monocalypt species (members of subgenus *Eucalyptus*) to have abundant flavanones (a class of flavonoid) in their leaves [5,6,7]. *Eucalyptus* flavanones can be *O*- and/or *C*-methylated (Figure 1) and flavanone profiles appear to be species-specific, making selected species attractive targets for both commercial extraction and use as model systems in which to study the molecular mechanism of flavonoid methylation. Nonetheless, little is known about the specific mechanism(s) of in planta *O*- or *C*-methylation in *Eucalyptus* or the biosynthesis of flavanones more broadly.

Recently, *Eucalyptus* species with high levels of the *O*-methylated flavanone pinostrobin (5-hydroxy-7-methoxy flavanone; Figure 1) and only very low levels of other methylated flavanones have been identified [5]. The biological role of pinostrobin in plants is not known, but more broadly, flavonoids protect plants from biotic stresses such as herbivores and pathogens, and a range of abiotic stresses including frost, drought and UV [8]. Pinostrobin has potential commercial value due to its numerous pharmacological and functional food benefits, including antiviral, antioxidant and anti-inflammatory activities and inhibition of intestinal smooth muscle contractions and sodium/calcium channel signalling pathways [4]. This study aims to identify and characterise the methyltransferase(s) involved in the regiospecific ornamentation of pinostrobin in *Eucalyptus* and thereby add to our limited understanding of flavanone biosynthesis in plants.

## 2. Results

### 2.1. Flavanone Profiling of Various Eucalyptus Species

**Figure 2:**
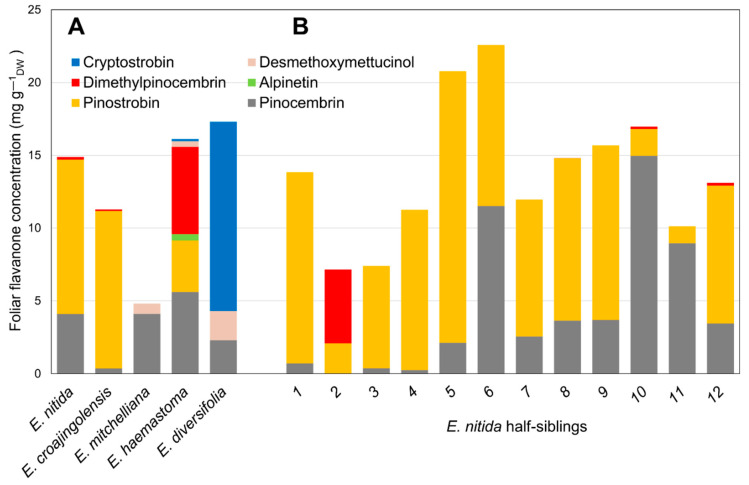
Foliar flavanone profiles of sapling cohorts of *Eucalyptus* species growing in a trial plantation. (**A**) Average data from 12 half-sibling saplings of 5 selected species; (**B**) individual data from half-siblings of *E. nitida*, the species chosen for transcriptome sequencing.

Based on the results of screens of subgenus *Eucalyptus* [5,6], half-sibling families of selected species with particular flavanone profiles were sown in a trial plantation. Twelve individual saplings from each of the five species (*Eucalyptus croajingolensis, E. diversifolia, E. haemastoma, E. mitchelliana* and *E. nitida*) were analysed for their foliar flavanone contents and their average flavanone profile was calculated (Figure 2A). The 7-*O*-methylated pinostrobin dominated the flavanone profiles of *E. croajingolensis* and *E. nitida* (Figure 2A), accounting for 95% and 72% of average total flavanones, respectively. Foliar extracts of *E. croajingolensis* contained a slightly higher average concentration of pinostrobin (10.8 mg g^−1^ dw) compared to that of *E. nitida* (10.6 mg g^−1^ dw). Samples from both species also contained the non-methylated flavanone pinocembrin and low levels of the di-*O*-methylated flavanone dimethylpinocembrin (Figure 2A). The initial extraction protocols used produced higher quality RNA from *E. nitida*; therefore, it was selected as the candidate species for the bulk of the transcriptome analysis and subsequent molecular characterisation work.

### 2.2. Isolation of O-methyltransferase from E. nitida

**Figure 3:**
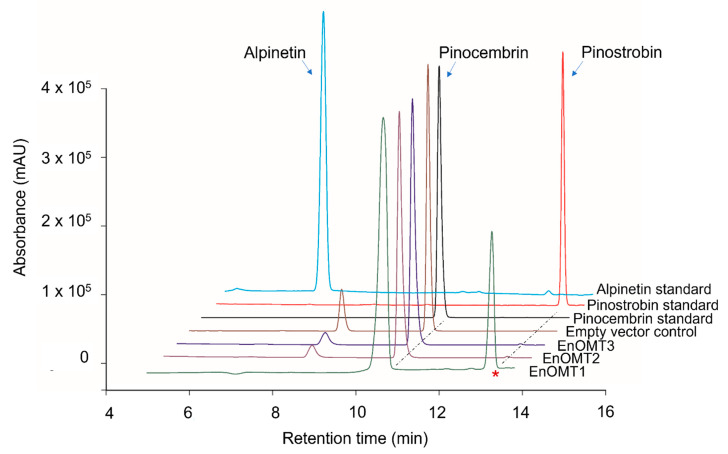
**High-performance liquid chromatography** (HPLC) chromatograms showing assay products of EnOMT1-3 with pinocembrin as substrate aligned with empty vector control and flavanone standards. The asterisk denotes the peak corresponding to the methylated assay product.

To isolate *O*-methyltransferases (OMTs) from *Eucalyptus*, transcriptome sequencing of *E. nitida* leaf tips was performed. Following RNA extraction and transcriptome assembly, over 60,000 unigenes were screened from the *E. nitida* leaf tip transcriptome, and approximately 60 were identified as potential *O*-methyltransferases based on sequence characteristics. Many methyltransferases exhibit a highly conserved C-terminal sequence, which was utilised for the selection of putative flavanone OMTs [9,10]. Unigene clusters that were annotated as methyltransferases were sorted based on their fragments per kilobase of transcript per million mapped reads (FPKM) value, which correlates directly with their expression levels in leaf tips (Figure S1). Three *OMT* candidates were selected (named *EnOMT1–EnOMT3*), based on their relatively high expression levels and sequence similarity to known *OMT* genes. The three *OMT* candidate sequences were amplified and cloned from *E. nitida* leaf tip cDNA using PCR-based methods, and were cloned in the protein expression vector pHUE for recombinant expression in *E. coli*. Enzyme extracts were incubated with pinocembrin as a substrate to test the EnOMT candidates for flavanone methyltransferase activity, using *S*-adenosylmethionine (SAM) as the methyl donor. Among the three candidates, HPLC analysis showed that one (EnOMT1) was able to successfully convert pinocembrin into a product with a later retention time than that of pinocembrin (Figure 3). Control assays (lacking substrate or with cell lysate of an expressed empty vector) were performed and showed no such activity.

To distinguish between the three possible *O*-methylated forms of pinocembrin (monomethylated pinostrobin and alpinetin, or dimethylated dimethylpinocembrin), identification of the assay product catalysed by EnOMT1 was achieved using (ESI)-LC-MS/MS analysis of the reaction mixture. Mass spectra of the control assay showed pinocembrin eluting at 11.8 min with *m*/*z* 257 [M+H]^+^, whereas the EnOMT1 assay product eluted at 14.3 min with *m*/*z* 271 [M+H]^+^ (Figures S2 and S3). The increase of 14 units corresponds to the addition of one methyl group in exchange for a proton, suggesting that the product formed is a monomethylated form of pinocembrin. The MS2 fragmentation pattern (Figures S2 and S3) suggests that methylation has occurred on the A-ring of pinocembrin; thus, the product can be either alpinetin (5-*O*-methylated) or pinostrobin (7-*O*-methylated). Since fragmentation across the A-ring is rarely observed, MS2 data alone are insufficient to differentiate between 5-*O*- and 7-*O*-methylation. Nonetheless, the retention time of the assay product matches that of an authentic standard of pinostrobin, suggesting it is 7-*O*-methylated. Moreover, HPLC analysis of the assay product spiked with the pinostrobin standard produced only one co-eluting peak. In addition, the UV absorption maxima of the assay product and the pinostrobin standard were both 288 nm, whereas that of the alpinetin standard was 285 nm, matching literature values of the two flavanones [11]. Therefore, it can be concluded that EnOMT1 catalysed the formation of pinostrobin, the 7-*O*-methylated derivative of pinocembrin.

### 2.3. Kinetic Analysis of EnOMT1

**Figure 4:**
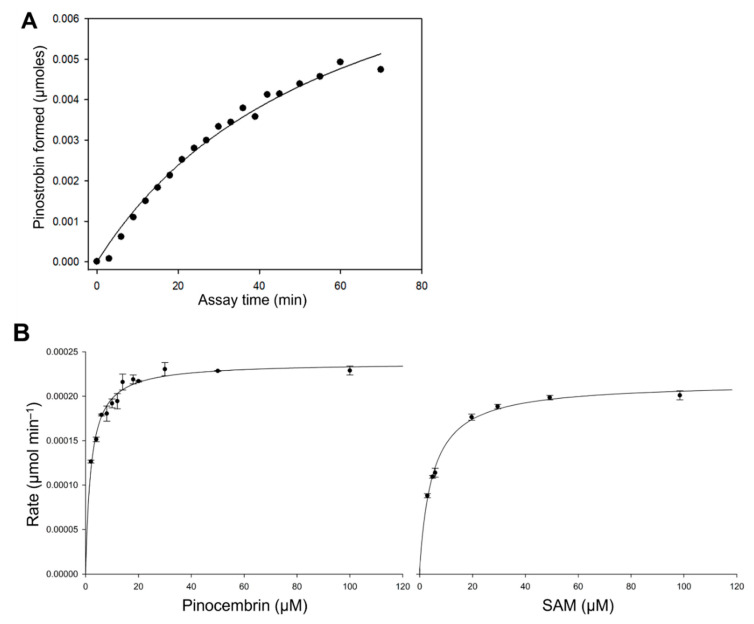
Kinetic characterisation of EnOMT1 methyltransferase. (**A**) Rate of formation of pinostrobin catalysed by EnOMT1 at a pinocembrin substrate concentration of 300 μM with respect to assay time; (**B**) Michaelis–Menten curves of pinostrobin produced by EnOMT1 over a range of pinocembrin and SAM concentrations.

A series of in vitro activity assays was performed with crude enzyme extracts to characterise the kinetic properties of EnOMT1. The rate of formation of pinostrobin by EnOMT1 enzyme extract in the presence of 300 μM pinocembrin is shown in Figure 4A, indicating that activity is maintained for up to 20 min under assay conditions. EnOMT1 catalytic velocity estimated at a series of substrate concentrations (Figure 4B) shows that the enzyme closely follows Michaelis–Menten kinetics [12].

Apparent kinetic constants were calculated due to the enzyme assays being conducted with crude or only partially purified enzymes. With an excess SAM concentration (100 μM) and varying quantities of pinocembrin as a substrate, EnOMT1 showed an apparent Km value of 2.05 ± 0.16 μM. At a saturating concentration of pinocembrin (50 μM), EnOMT1 showed an apparent Km of 4.99 ± 0.29 μM for SAM. The specific activity of the enzyme (U mg protein^−1^) and catalytic rate constant (kcat) were calculated by performing a short assay with a substrate concentration at Km app using an enzyme purified through His-tag affinity chromatography. The affinity-purified fraction of EnOMT1 showed specific activity of 0.0033 U mg^−1^ protein and had a catalytic rate constant (kcat) of 0.1 s^−1^ and catalytic efficiency (kcat/Km) of 0.05 μM^−1^ s^−1^.

**Figure 5:**
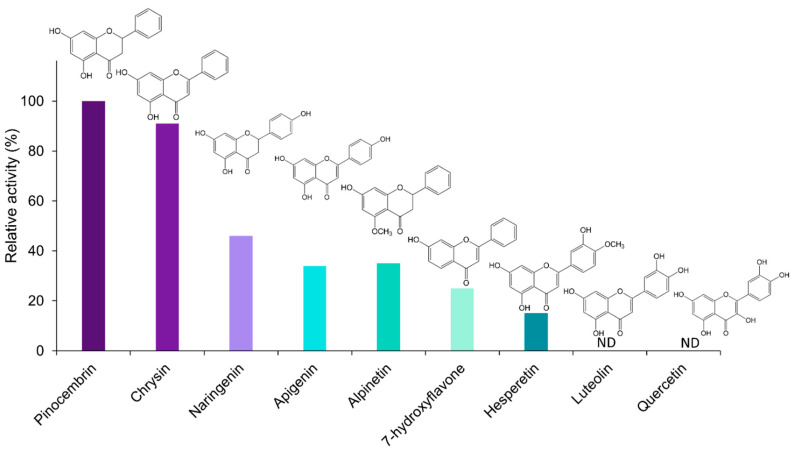
Relative activity (%) of EnOMT1 with various flavonoid compounds as substrate. Data were normalised to pinocembrin activity of 100%. All substrates were at 10 μM and a saturating SAM (500 μM) was used in all assays. ND = activity not detected.

The catalytic specificity of EnOMT1 was analysed against a range of related flavonoid and phenolic compounds in order to obtain substrate preference and apparent kinetic data. All flavonoids tested are known to be present in the genus *Eucalyptus* [13,14]. With SAM as a methyl donor, EnOMT1 was shown to possess activity against the flavanones alpinetin, naringenin and hesperetin and the flavones 7-hydroxyflavone, apigenin and chrysin (Figure 5). Among the flavonoid compounds tested, no activity was observed with 5-hydroxyflavone, 6-hydroxyflavone, luteolin or quercetin. 

### 2.4. Isolation of OMT1 in Other Eucalyptus Species

**Figure 6:**
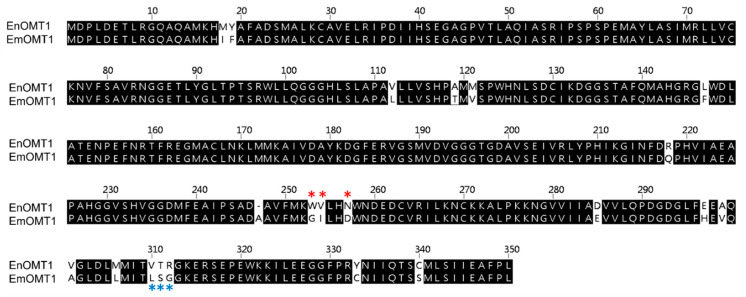
Amino acid alignment of OMT1 sequence from *E. nitida* (EnOMT1) and *E. mitchelliana* (EmOMT1). * Variable amino acids of Region 1 are indicated by red asterisks, while variable amino acids of Region 2 are indicated by blue asterisks.

To determine whether the *OMT1* gene identified in *E. nitida* also plays a role in shaping the varying flavanone profiles observed in other *Eucalyptus* species, the equivalent *OMT1* gene from species of contrasting flavone profiles was investigated. Using a PCR-based approach, the *OMT1* gene from *E. croajingolensis* (which possesses high pinostrobin content, similar to *E. nitida*) and *E. mitchelliana* (which accumulates pinocembrin and lacks pinostrobin; Figure 2A) was amplified and sequenced. The OMT1 sequence identified in *E. croajingolensis* (hereafter named EcOMT1) is almost identical to that of EnOMT1, displaying 99% amino acid identity (Figure S4), while the OMT1 sequence in *E. mitchelliana* (EmOMT1) displays 94% amino acid identity to EnOMT1 (Figure 6).

### 2.5. Gene Expression of OMT1 in E. nitida and E. mitchelliana 

**Figure 7:**
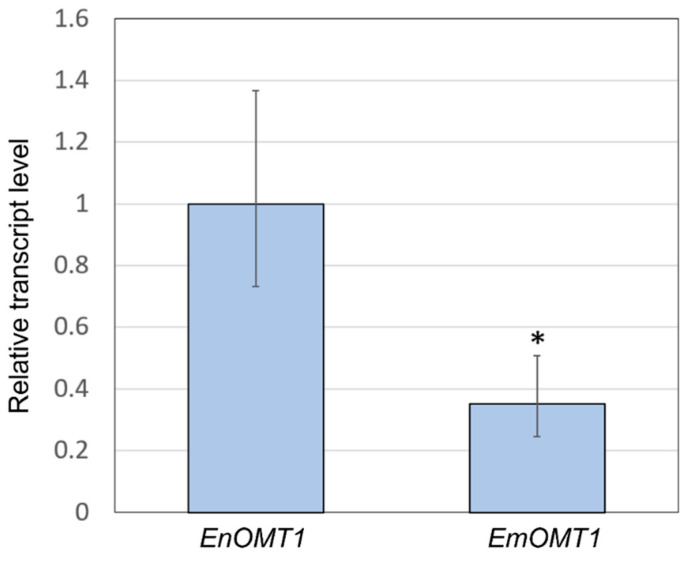
**Quantitative polymerase chain reaction** (qPCR) analysis demonstrating relative transcript level of the OMT1 gene in *E. nitida* (*EnOMT1*) and *E. mitchelliana* (*EmOMT1*). *EnOMT1* transcript level is set to 1. Error bars indicate standard deviation. Asterisk (*) indicates statistically significant difference compared to EnOMT1 (*p* < 0.005).

The relatively high amino acid sequence identity between EnOMT1 and EmOMT1 prompted investigation into whether a difference in *OMT1* gene expression might account for the lack of pinostrobin in *E. mitchelliana*. Primers designed for a conserved region (Table S1) were used in qPCR gene expression analysis with *E. nitida* and *E. mitchelliana* cDNA derived from leaf tips as template. Expression of *EmOMT1* in *E. mitchelliana* leaf tips was found to be reduced to approximately one-third of the level of that of *EnOMT1* in *E. nitida* leaf tips (Figure 7). Although the difference in expression is significant (*p* < 0.005), it does not appear likely that this level of reduction in transcript would be solely responsible for the complete lack of pinostrobin observed in *E. mitchelliana* leaves. Thus, the catalytic activity of EmOMT1 was investigated. 

### 2.6. Activity of OMT1 Homologues in Additional Eucalyptus Species 

The full-length sequence of EmOMT1, along with that of EcOMT1, was cloned, recombinantly expressed in *E. coli* BL21 cells and assayed for activity using pinocembrin as a substrate in an identical manner as described for EnOMT1. As expected, EcOMT1 exhibited an almost identical conversion of pinocembrin to pinostrobin with an apparent Km app of 2.55 ± 0.38 μM (Figure S5). Conversely, EmOMT1 was found to require a much longer (16 h) incubation period to allow for formation of the methylated product pinostrobin compared to EnOMT1 (3 min for the equivalent amount of pinostrobin formation by EnOMT1). An extrapolated non-linear regression analysis showed that the apparent Km of EmOMT1 is greater than 800 μM and the activity of EmOMT1 is two orders of magnitude less than that of EnOMT1.

### 2.7. Analysis of Critical Residues of EnOMT1 

**Figure 8:**
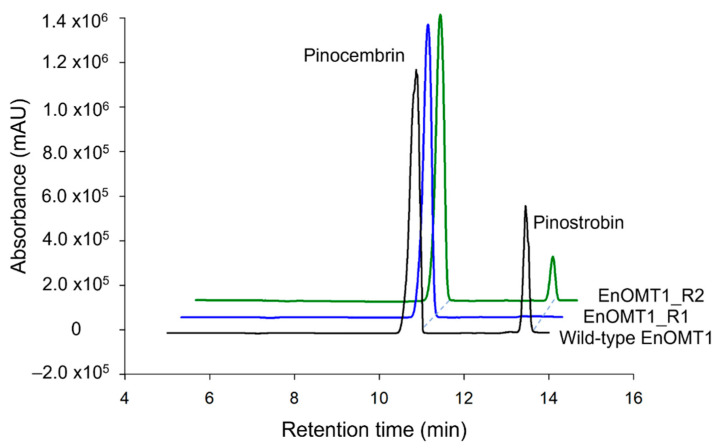
HPLC chromatograms of EnOMT1 mutant assay products. Each assay was incubated with 50 μM pinocembrin as substrate for 10 min.

The high sequence similarity between EnOMT1 and EmOMT1, combined with the stark difference in their methyltransferase activity, presents an opportunity to identify residues which may be critical for EnOMT1 activity. Therefore, regions which displayed sequence variation between EnOMT1 and EmOMT1 were analysed for further investigation. Two regions in particular emerged as candidates for being important for enzyme activity. Region 1 and 2 (Figure 5) contain amino acids potentially involved in SAM binding and substrate binding, respectively, and the equivalent amino acids from these regions in previously studied plant OMTs have been shown to impact catalytic activity [15,16]. Using primer-based site-directed mutagenesis techniques, the three variable amino acids in each region of EnOMT1 were mutated towards the EmOMT1 sequence (Region 1: W252G, V253I, N256D and Region 2: V309L, T310S, R311G). EnOMT1 with mutations in Region 1 (termed EnOMT1_R1) and Region 2 (termed EnOMT_R2) were cloned into pHUE and expressed in BL21 cells to be assayed in the presence of pinocembrin for enzyme activity. In vitro activity assays with EnOMT1_R1 demonstrated loss of catalytic activity, with no pinostrobin detected, while EnOMT1_R2 demonstrated reduced catalytic activity, with pinostrobin being detected at a lower level compared to that of wild-type EnOMT1 (Figure 8). 

**Figure 9:**
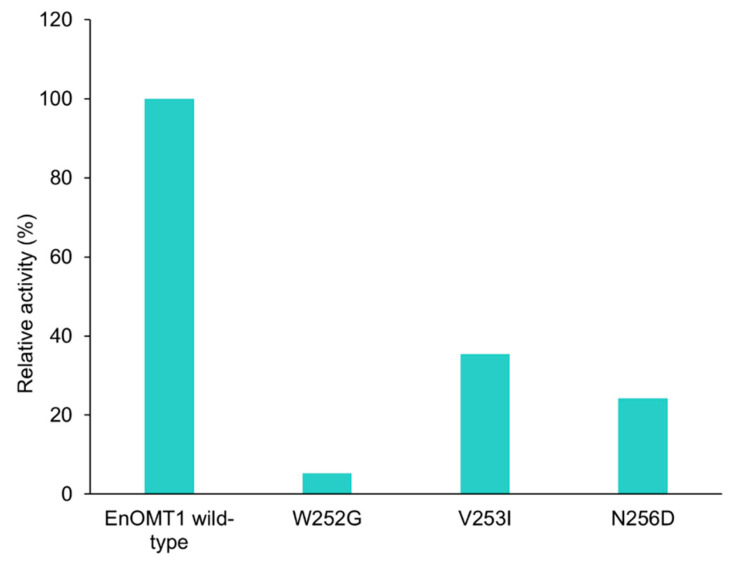
Activity of EnOMT1 single mutant clones (W252G, V253I and N256D) compared to that of wild-type EnOMT1, expressed in terms of the percentage of pinocembrin converted into methylated product.

To further investigate the amino acids in Region 1, each individual variant residue was mutated and tested separately. Of the three amino acid changes, W252G displayed the strongest effect, resulting in a 95% reduction in methyltransferase activity in comparison to wild-type EnOMT1. V253I and N256D also demonstrated an effect on catalytic activity, resulting in 65% and 75% reduction in activity, respectively (Figure 9). 

## 3. Discussion

The diversity of methylated flavonoids found in *Eucalyptus* species provides an ideal system for identifying the specific methyltransferases involved in the chemical ornamentation of flavonoids. Utilising this diversity, we identified a species (*E. nitida*) which contains a high level of the *O*-methylated flavanone pinostrobin, and through a transcriptomic approach were able to identify a gene (*EnOMT1*) expressed highly in the expanding leaf tips encoding an *O*-methyltransferase protein which acts on the unsubstituted B-ring flavanone, pinocembrin. The isolated EnOMT1 sequence encodes a 349 amino acid protein with sequence similarity to prenylchalcone OMTs, alkaloid OMTs and other known flavonoid OMTs [17,18,19]. EnOMT1 is the first flavanone methyltransferase shown to be acting on pinocembrin, and the first methyltransferase characterised from *Eucalyptus*.

The kinetic properties of EnOMT1 (Km = 2.05 ± 0.16 μM, kcat of approximately 0.1 s^−1^) are comparable to the published values observed for the isoflavone 4′OMT from garden pea (*Pisum sativum*; Km = 3 μM for 2,7,4′-trihydroxyisoflavanone; [20]), naringenin 7-OMT from rice (*Oryza sativa*; Km = 1.9 ± 0.1 μM for naringenin [21]) and a flavonol OMT from *Chrysosplenium americanum* (Km = 6.4 μM for 3′,5-dihydroxy-3,4′,7-trimethoxyflavone; [22]). Moreover, they are in very close agreement with an improved variant of *Thalictrum flavum* (S)-scoulerine 9-OMT (Km for alkaloid (S)-scoulerine of 2.91 ± 1.20 μM and a kcat of 0.10 ± 0.01 s^−1^) recently produced by structure-guided engineering of the wild-type enzyme [23]. The EnOMT1 Km value of 4.99 μM for SAM is moderately high, but lower than most previously characterised OMT enzymes [15,24,25]. Given the relatively high concentrations of pinostrobin found in *E. nitida* (Figure 2), it is unsurprising that EnOMT1 possesses a high affinity for both its substrates, pinocembrin and SAM, thereby achieving high levels of conversion. 

The in vitro substrate specificity studies presented here demonstrated that only flavonoid substrates with a free hydroxyl at position 7 on the A-ring were methylated by EnOMT1 (albeit to different degrees), indicating EnOMT1 is a 7-OH-specific methyltransferase of flavonoids. Such stringent regiospecificity has been described as a general attribute of plant flavonoid 7-OMTs [21,26,27]. The 7-OH is much more acidic than 5-OH due to the strong hydrogen bonding between the carbonyl oxygen at position 4 and the hydrogen of the 5-OH [28,29]. Overcoming the hydrogen bonding to deprotonate and methylate the 5-OH is likely to require a methyltransferase with a different mode of action to 7-OH methyltransferases.

Surprisingly, no products were obtained from assays of EnOMT1 with the 7-OH substrates quercetin and luteolin. Both quercetin and luteolin possess two B-ring hydroxyls, suggesting that the presence of these moieties may inhibit EnOMT1 activity. The majority of the flavonoid *O*-methyltransferases characterised to date accept quercetin as a substrate and show a specific pattern in the methylation of hydroxyls on the meta (6-, 8-, 3′-), para (7-, 4′-) and 3-positions of the flavonoid ring system [30]. The C-ring hydroxyl of quercetin is known to influence substrate binding, and the substitution of its 3-OH affects enzyme activity [31,32]. Methylation of 3′-OH is a prerequisite for 7-OH as well as B-ring methylation of quercetin in *Chrysosplenium americanum* flavonoid glucoside biosynthesis [20]. Therefore, it is possible that the 3-OH and/or 3′-OH of quercetin may interfere with EnOMT1 substrate binding. 

The reduction of EnOMT1 activity with apigenin and hesperetin and its complete absence with luteolin and quercetin demonstrates the drastic effect B-ring substitutions have on the activity of the enzyme. Consistent with the results from studies of other flavonoid OMTs, this study further strengthens the relationship between a substrate’s hydroxylation pattern and its methyl-accepting ability. In *C. americanum*, the nucleophilicity of A-ring hydroxyls and, consequently, their reactivity towards *O*-methylation are affected by B-ring substitution [20]. Similarly, a 4′-O-methyltransferase isolated from *C. roseus* only methylated flavonoids with the same B-ring hydroxyl configuration as that of its substrate homoeriodictyol (3′-methoxy and 4′-hydroxyl) and a specific A-ring substitution pattern [33]. In the case of EnOMT1, substrates with only 4′-OH on the B-ring showed a reduced level of activity, but no activity was detected for compounds with vicinal hydroxyl groups on the B-ring. In a study of in vitro *O*-methylation, cell-free extracts of calamondin orange tissues catalysed methylation of a number of hydroxyls on quercetin and its derivatives [30]). The authors consequently suggested that various interactions of A- and B-ring meta (6-, 8-, 3′-) and para (7-, 4′-) hydroxylation patterns influence methylation. The rigid para-quinoid structure, which results from hydroxylation at the 4′-position, results in resonance of the flavonoid skeleton and thus a limit to A-ring methylation when there is no vicinal hydroxyl in position 6′- or 8′- [30]. Therefore, the lack of hydroxylation at the 6′- and 8′- positions of the flavonoid structures tested in this study might account for the reduced activity of EnOMT1 towards substrates with 4′-OH methylation. Substrate hydroxylation at both 3′- and 4′- positions completely removed activity of EnOMT1 towards the A-ring 7-OH in the compounds examined here. Even though EnOMT1 did not accept luteolin, hesperetin (a monomethylated derivative of luteolin at 4′-OH) showed approximately 15% activity compared to pinocembrin (Figure 5). Thus, it appears the methylation of the 4′-OH enables EnOMT1 to further methylate hesperetin at the 7-OH position. The degree of methylation of the functional hydroxyl groups is known to regulate the biosynthesis of various secondary metabolites [34,35]. Therefore, the methylation of the 4′-OH appears to have enabled EnOMT1 to accommodate hesperetin into its active site.

It is noteworthy that EnOMT1 acted upon alpinetin, a 5-*O*-methylated flavanone present at low levels in some *Eucalyptus* species, converting it to the di-*O*-methylated dimethylpinocembrin. Nonetheless, the relative activity of EnOMT1 with alpinetin as a substrate was approximately 30% compared to pinocembrin. The two *Eucalyptus* species analysed in this study possessing a high concentration of 7-*O*-methylated pinostrobin (*E. nitida* and *E. croajingolensis*) failed to show any detectable amount of alpinetin, but do accumulate appreciable amounts of dimethylpinocembrin (Figure 2). It is possible that the highly active EnOMT1 immediately converts any alpinetin that is present in cells to dimethylpinocembrin, which is then transported to and sequestered in the foliar secretory glands.

Multiple methylations of flavonoids are usually catalysed by distinct enzymes [20]; however, sequential methylation of flavonoids by the same enzyme has been observed previously [36]. Particularly, CdFOMT5 from *Citrus depressa* methylates four different hydroxyl positions on quercetin A- and C-rings in no particular sequence [32]. In contrast, TaOMT2 from wheat exhibits strict sequential methylation of B-ring 3′-, 4′- and 5′-OH of tricetin proceeding in an order of 3′-mono-→3′,5′-di-→3′,4′,5′-trimethyl ether derivatives [37]. However, in this study, no evidence of sequential methylation of pinocembrin by EnOMT1 was found. Therefore, a separate flavanone OMT, specifically methylating an A-ring 5-hydroxyl on pinocembrin, is expected to be involved in the synthesis of alpinetin and dimethylpinocembrin.

The search for an equivalent *OMT1* gene in *E. mitchelliana*, a species which accumulates no methylated flavanones despite containing a moderately high quantity of pinocembrin in foliar glands, led to the isolation of a gene encoding a protein (EmOMT1) with high sequence identity with EnOMT1 (94% amino acid identity), but very low methyltransferase activity. While this difference in activity may explain the lack of pinostrobin observed in *E. mitchelliana*, the high sequence similarity between EmOMT1 and EnOMT1 also provided an opportunity to investigate the amino acids which may be critical for the activity of EnOMT1, in combination with sequence data from previous studies characterising *O*-methyltransferases. For example, an investigation into a hydroxyisoflavanone 4′-O-methyltransferase in *Medicago truncaluta* [15] identified active residues of Mt HiOMT, including those structurally equivalent to W252, N256, V309 and T310 of EnOMT1, amino acids which vary between EnOMT1 and EmOMT1 (Figure 5). Similarly, a study of a caffeic acid *O*-methyltransferase in ryegrass found the equivalent of N256 (D267 in LpOMT1) to be a catalytic residue critical for hydrogen bond interactions [16]. The same study also identified amino acids at the equivalent positions of EnOMT1 V309 and T310 as being important in promoting cavity formation necessary for substrate binding. While unchanged between EnOMT1 and EmOMT1, the histidine at position 255 of EnOMT1 has also been found to be a critical residue for activity in several OMTs, furthering the notion that the cluster of amino acid variations in Region 1 (aa 252–256), in addition to those in Region 2 (aa 309–311), might contribute to the reduction in activity of EmOMT1 [16,19,38,39,40]. Site-directed mutagenesis of EnOMT1 to mimic the sequence changes of the inactive EmOMT1 in Regions 1 and 2 demonstrated that the mutations in Region 1 caused a severe loss of methyltransferase activity, with no pinostrobin being detected during in vitro assays with EnOMT_R1 using pinocembrin as the substrate. Further site-directed mutagenesis analysis of the individual residues within Region 1 demonstrated that the mutation W252G was sufficient to eliminate 95% of activity in vitro, highlighting the critical role of this amino acid, and to a lesser extent that of Val253 and Asn256. It appears, therefore, that in addition to the catalytic His255 residue, the adjacent residues play critical associative roles, possibly forming a channel for methyl passage [38]. 

It is likely that the amino acid changes applied in this study affect either the potential of OMT1 to bind SAM, or to bind the pinocembrin substrate. The amino acid at the structural equivalent of V309 has previously been suggested to be important for cavity size for flavone binding [26], and thus the changes from EnOMT1 observed in Region 2 (aa 309, 310, 311) in EmOMT1 could result in reduced cavity space for binding of pinocembrin. The OMT1 sequence in *E. croajingolensis* was found to be almost identical to that of EnOMT1 and is functionally active, correlating with the high pinostrobin content observed in *E. croajingolensis*, adding further credence to the notion that the functional status of OMT1 in *Eucalyptus* species is a critical determinant of their flavanone profile. 

The efficiency of OMT1 in various *Eucalyptus* species may have a particularly strong influence on the observed flavanone profile due to substrate sequestration before the enzyme can gain access. Two biosynthetic enzymes, chalcone synthase and chalcone isomerase, responsible for the production of flavanones, have been shown to be located not only in the cytoplasm but also in the nuclei of some cells [41]. This differential localisation of enzyme systems may be a conserved mechanism for controlling the site-specific accumulation of flavonoid end products. In *Eucalyptus*, the flavanone pinocembrin and its methylated derivatives are localised to the extracellular lumen of subdermal secretory glands in the leaf [42]. The methyltransferase enzymes, on the other hand, are known to be a component of an aggregated multienzyme system on the surface of the endoplasmic reticulum within cells [20]. Tabersonine 16-*O*-methyltransferase encoded by 16OMT in *C. roseus* has been localised specifically in abaxial and adaxial epidermal cells of young leaves, and GFP imaging analysis showed that the enzyme is organised in vivo as a homodimer in the cytosol of these epidermal cells [43]. Hence, from a physiological point of view, it is possible that the metabolic channelling of pinocembrin into the gland lumen would prevent its efficient uptake by the cytosolic or ER-anchored OMT1. The enzymes involved in plant-specialised metabolism often have low turnover numbers [44,45,46]. To overcome this lower catalytic efficiency, plants often express such enzymes at very high levels in the appropriate tissue and cell type to achieve the required level of catalytic activity [47]. qPCR analysis shows that the expression level of *EmOMT1* is approximately one-third of that of *EnOMT1* (Figure 7). This reduced expression, along with the reduced activity of the EmOMT1 enzyme combined with pinocembrin sequestration to the gland lumen immediately after synthesis, likely explains the absence of any detectable amounts of methylated derivatives of pinocembrin in *E. mitchelliana* gland extracts.

## 4. Materials and Methods

### 4.1. Plant Material and Flavanone Extraction

Fully expanded leaves were collected from *Eucalyptus* saplings growing in a trial plantation at The University of Melbourne’s Dookie Campus, Victoria, Australia (36°23′3″ S, 145°42′52″ E) and dried for 1–2 days at 55 °C. Dried leaves were finely ground and extracted with 4 mL acetonitrile (100%) in a previously weighed glass vial. Samples were agitated on an Intelli mixer (99 rpm, room temperature) for 24 h. The extract was then centrifuged (5000× *g*, 5 min) to remove leaf residue and the supernatant filtered through a syringe filter (PTFE membrane, 0.45 μm pore size) before HPLC analysis. Vials were left in the fume hood to evaporate the residual solvent, dried at 55 °C and weighed accurately to calculate the leaf dry weight. 

### 4.2. RNA Isolation

RNA was isolated from leaf tips of *E. nitida*, *E. mitchelliana* and *E. croajingolensis* (100 mg fresh weight) following the methods described at http://www.untergasser.de/lab/protocols/miniprep_rna_ctab_v1_0.htm (accessed on 15 March 2022). Following isopropanol precipitation, RNA was purified using the RNeasy mini spin column from RNeasy Plant mini kit (QIAGEN) following the manufacturer’s instructions. Contaminating DNA was removed via DNase1 (Invitrogen, Carlsbad, CA, USA) treatment following manufacturer’s instructions. cDNA was synthesised from 2 μg of RNA using SuperScript™ III Reverse Transcriptase (Invitrogen) in a total volume of 20 μL, following manufacturer’s instructions, with the only modification being that the reaction was incubated at 25 °C for 10 min, 37 °C for 45 min, 42 °C for 45 min and 70 °C for 15 min.

### 4.3. Transcriptome Sequencing

Purified total RNA was processed by Novogene for transcriptome sequencing. The NEBNext^®^ Ultra™ II RNA Library Prep Kit for Illumina^®^ (New England Biolab Inc., Ipswich, MA, USA) was employed to convert RNA into high-quality non-directional libraries for next-generation sequencing on the Illumina^®^ platform. The original raw data from Illumina HiSeq 2500 platform were transformed to sequenced reads by the base calling method, generating150 bp paired end reads. Raw reads were filtered to remove reads containing adapters or reads of low quality (reads where uncertain nucleotides constitute more than 10% of either read or where low-quality nucleotides constitute more than 50% of the read). Clean reads were de novo assembled for transcriptome reconstruction using the bioinformatic platform Trinity (version r20140413p1, *k*-mer size = 25. Minimum *k*-mer coverage = 2; [48]). Putative *O*-methyltransferases in the assembled transcriptomes were identified via database BLAST search (Geneious Prime Version 2019.0.4; Biomatters Ltd., Auckland, New Zealand) using known *O*-methyltransferase sequences from various species.

### 4.4. Recombinant Protein Expression

Full-length coding sequences of putative OMTs were amplified using primers listed in Table S1. PCR products were digested by the appropriate enzyme (restriction sites incorporated into the primer sequences) and ligated into the multiple cloning site of the pHUE expression vector [49]. The resulting plasmids were confirmed by restriction digest and sequencing, then transferred into *Escherichia coli* BL21 (DE3). BL21 cells containing the expression vectors were grown in 200 mL volume LB at 37 °C until OD_600_ = 0.6, and protein production was induced by addition of 0.1 mM isopropyl β-D-1-thiogalactopyranoside (IPTG) at 16 °C for 16 h. The cell pellet was resuspended in lysis buffer (50 mM NaH_2_PO_4_, 300 mM NaCl, 10 mM imidazole, protease inhibitor cocktail (Roche, Basel, Switzerland), 1 mg ml^−1^ Lysozyme), homogenised by gentle mixing at room temperature for 30 min and sonicated to release the intracellular contents. The resulting solution was then centrifuged at 10,000× *g* for 5 min, and the supernatant was used as a crude enzyme solution for the enzyme assays. To confirm expression, crude extracts were fractionated by SDS-PAGE and transferred to a nitrocellulose membrane using a Bio-Rad transfer apparatus. After blocking with 3% non-fat milk in TBST (10 mM Tris, 150 nM NaCl, 0.5% Tween-20), the membrane was probed with an antibody against the His tag (Life Technologies, Roskilde, Denmark, 1 μg mL^−1^). Following three TBST washes, blots were incubated with a 1:10,000 dilution of horseradish-peroxidase-conjugated anti-mouse antibody and developed with the SuperSignal West Femto Maximum Sensitivity Substrate following manufacturer’s instructions (Thermo Fisher, Waltham, MA, USA).

### 4.5. Site-Directed Mutagenesis

Site-directed mutagenesis of EnOMT1 was achieved by a primer extension method. Primers containing the desired mutations were designed towards three amino acids within Region 1 and Region 2, and also individual residues within Region 1 (Table S1), which were used in conjunction with the EnOMT1F and EnOMT1R primers in separate PCR reactions to create two overlapping fragments that were purified and combined to be used as template for a subsequent PCR reaction with the EnOMT1_F and EnOMT1_R primers. This generated the sequence to be cloned into the pHUE vector.

### 4.6. Enzyme Assay and Kinetics

The activity of crude OMT was assayed with various substrates, following the protocol described by Itoh et al. (2016), in 500 μL reactions containing 50 mM potassium phosphate buffer (pH 7), 200 μL crude OMT enzyme (approx. 0.6 mg protein), 500 μM SAM and 10–100 μM substrate (dissolved in DMSO). Several negative controls (various combinations lacking enzyme, substrate or SAM) were run along with the test to account for the accuracy of the analysis. Equivalent amounts of crude enzyme preparations were examined by Western blot analysis to confirm expression of each His-tagged OMT (Figure S6). All reactions were incubated at 30 °C with gentle shaking for 20 min and terminated by the addition of 500 μL acetonitrile. The soluble fraction of the assay mixture, containing the methylated products, was separated from the precipitate by centrifugation and analysed using RP-HPLC. Enzyme kinetic parameters of EnOMT1 were calculated using pinocembrin as the substrate. The kinetic constants Vmax and Km of EnOMT1 were determined using the Enzyme Kinetics add-on programme in SigmaPlot software. The specific activity of the enzyme (U mg^−1^ protein) and catalytic rate constant (k_cat_) was calculated from the maximum catalytic velocity of purified EnOMT1.

For determination of substrate specificity of EnOMT1, a range of phenolic compounds (alpinetin, naringenin, hesperetin, 5-hydroxyflavone, 6-hydroxyflavone, 7-hydroxyflavone, chrysin, apigenin, luteolin, quercetin, pinocembrin chalcone, caffeic acid, *p*-coumaric acid, trans-cinnamic acid, catechol and myricitrin) were used as substrates in crude enzyme assays. Duplicate assays with the crude enzyme and varying substrate concentrations (2–50 µM) at a saturating concentration of 500 µM SAM were carried out for time intervals ranging from 3 to 10 min, where the reaction velocity is in the linear range with respect to time for EnOMT1 with pinocembrin as a substrate. Control reactions without EnOMT1 were run to determine the initial concentration of substrates. After RP-HPLC analysis, the peak area of the remaining substrate in the assay mixture was compared with that of the control and the difference in peak size area was used as the amount of substrate utilised during the reaction. For calculation of relative activity, the reactions at saturating concentrations (10 µM substrate, 500 µM SAM) were used. Relative activity was calculated by setting the amount of pinocembrin utilised by EnOMT1 as 100%.

### 4.7. Liquid Chromatography and Mass Spectrometry of Flavanones

Flavanone profiling of *Eucalyptus* leaves as well as chromatographic separation of the reaction mixture was achieved on an Agilent 1220 Infinity High-Performance Liquid Chromatography (HPLC) system with a Gemini C18 analytical column (5 μm, 150 × 4.6 mm; Phenomenex), Degasser G1311 A, pumpL7100, autosampler L-2700 and a photodiode array with UV detection (288 nm). A gradient elution method with water (solvent A) and acetonitrile (solvent B) at a flow rate of 1 mL min^−1^ was employed for flavanone profiling whereas both solvents were acidified (0.1% acetic acid) for enzyme assay product analysis. The column temperature was maintained at 28 ± 5 °C and the injected volume varied between 0.1 μL and 100 μL according to the sample′s composition. The elution method used was as follows: linear increase from 30% to 50% solvent B in 7 min, thereafter to 95% in the next 7 min, isocratic elution with 95% solvent B for 6 min, then a linear gradient of 95% to 100% solvent B over 15 s followed by 100% solvent B for 3.75 min.

Flavanones were identified by comparison with authentic compounds purchased from Sigma-Aldrich (St. Louis, MO, USA). Calibration series of pinostrobin and pinocembrin were measured at 288 nm (the absorbance maximum for pinostrobin). Series were performed in triplicate and linear regressions were fitted to the average data and used for quantification. The average pinostrobin calibration series had a slope value of 6.9 × 10^7^ (*p* < 0.0001, r^2^ = 0.999) whereas that of pinocembrin had a lower slope value of 5.5 × 10^7^ (*p* < 0.0001, r^2^ = 1.0). Values for all compounds other than pinocembrin are presented as pinostrobin equivalents. The limit of quantitation was set at a minimum peak area of 1000 mAU, which equates to 0.01 ng of pinostrobin. Where required, identification of enzyme assay products was performed using Electro-Spray Ionisation Liquid Chromatography-Mass Spectrometry (ESI-LCMS) on an Agilent 6520 quadrupole time of flight mass spectrometer system (TOF; Agilent Technologies, USA) following the method described in [5]. Accurate mass data calculation of the molecular ions, as well as the processing of chromatograms, was performed using Mass Hunter Workstation (Agilent Technology). A gradient elution method with water (solvent A) and acetonitrile (solvent B), both acidified with 0.1% acetic acid, was employed. The eluent system was a gradient of solvent B from 30 to 50% over 7 min, followed by 50–95% over 7 min and then isocratic at 95% for a further 12 min, and finally a gradient of 95–100% over 0.25 min. Electrospray ionisation was achieved in positive ion mode using the following conditions: nebuliser pressure 37 psi, gas flow rate 12 L min^−1^, gas temperature 350 °C, capillary voltage 4000 V, fragmentor 150 and skimmer 65 V. The conditions were modified depending on the compound to be analysed whenever needed, and other source parameters were set to standard conditions recommended by Agilent. TOF mass range was set from *m/z* 70–1700. 

### 4.8. Quantitative Real-Time PCR

cDNA was generated for each species from 2 μg triplicate RNA samples as described above. The *Eucalyptus* gene encoding a cyclin-dependent protein kinase (Eucons04) was selected as a reference gene based on de Oliveira et al. (2012). Quantitative real-time PCR (qPCR) primers (Table S1) targeting a region common to *EnOMT1* and *EmOMT1* were designed using the online PrimerQuest tool from Integrated DNA Technologies (https://sg.idtdna.com/Primerquest/Home/Index, accessed on 15 March 2022). qPCR was carried out using QuantiNova™ SYBR^®^ Green PCR kit following manufacturer’s instructions. Reactions were analysed using CFX384 Touch Real-Time PCR detection system (Bio-Rad, Hercules, CA, USA). Real time cycler program was set to 95 °C for 2 min for initial heat activation followed by 30 cycles of denaturation (5 s at 95 °C) and combined annealing/extension (60 °C for 10 s). Melting curve analysis was performed using the real-time cycler built-in program. Expression levels were calculated relative to those of *EnOMT1* in *E. nitida* using the associated CFX Maestro Software (Bio-Rad).

## 5. Conclusions

RNA-seq analysis of leaf tips enabled the isolation of a gene encoding a flavanone 7-*O*-methyltransferase (*EnOMT1*) from *Eucalyptus*. Biochemical characterisation of its in vitro activity revealed a range of substrates upon which EnOMT1 acts in a regiospecific manner. Comparison to a homologous sequence from a *Eucalyptus* species lacking *O*-methylated flavonoids identified critical catalytic amino acid residues within *EnOMT1* responsible for its activity. This detailed molecular characterisation identified a methyltransferase responsible for chemical ornamentation of the core flavanone structure of pinocembrin and helps shed light on the mechanism of flavanone biosynthesis in *Eucalyptus*.

## Data Availability

RNA-seq data have been deposited in the Sequence Read Archive at: https://www.ncbi.nlm.nih.gov/sra/SRR18080945 (accessed on 15 March 2022) (BioProject PRJNA808754). GenBank accession numbers for genes described in this study are as follows: *EnOMT1* (OM964910), *EnOMT2* (OM964911), *EnOMT3* (OM964912), *EmOMT1* (OM964909) and *EcOMT1* (OM964908).

## Supplementary Materials

The following supporting information can be downloaded at: https://www.mdpi.com/article/10.3390/ijms23063190/s1.
