# Supplementary material for: Molecular Characterisation of Flavanone O-methylation in Eucalyptus
Source: Int J Mol Sci. 2022 Mar 16;23(6):3190. doi: 10.3390/ijms23063190 (PMC8954846; doi:10.3390/ijms23063190)
Supplement: Supplementary file 1 [file ijms-23-03190-s001.zip › Supplementary Figures.pdf]

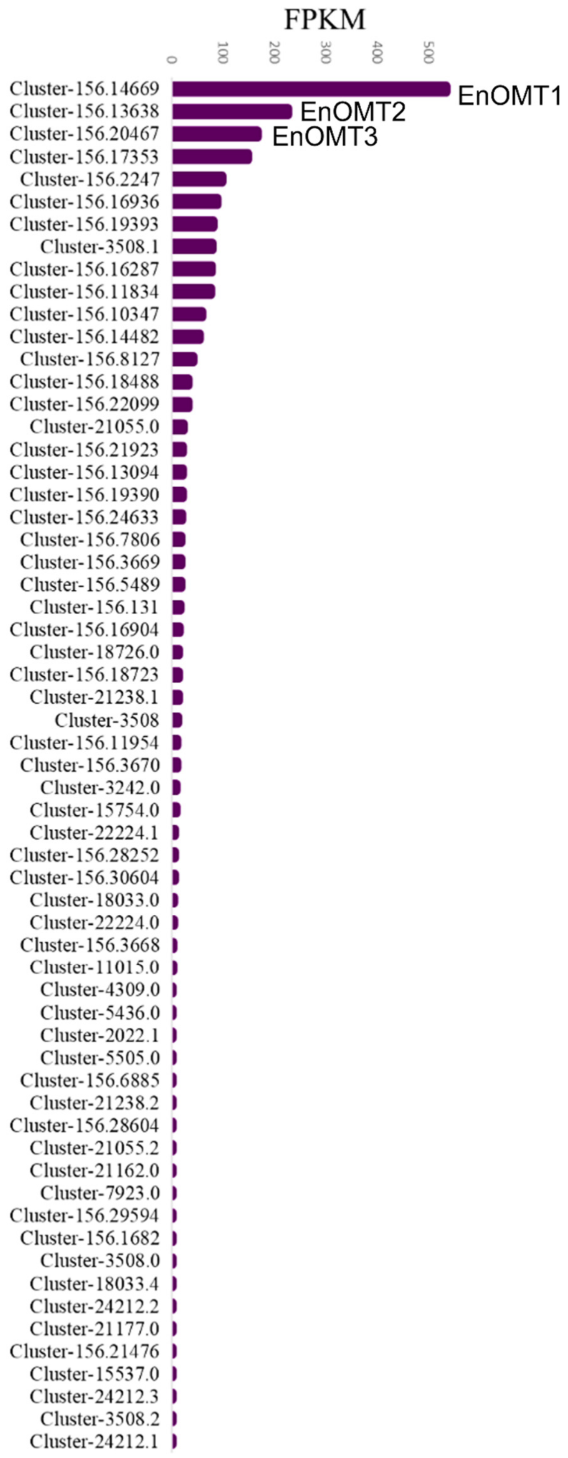

Figure S1. Unigene clusters annotated as methyltransferases in *E. nitida.* Clusters (X-axis) are presented with corresponding FPKM values (Y-axis).

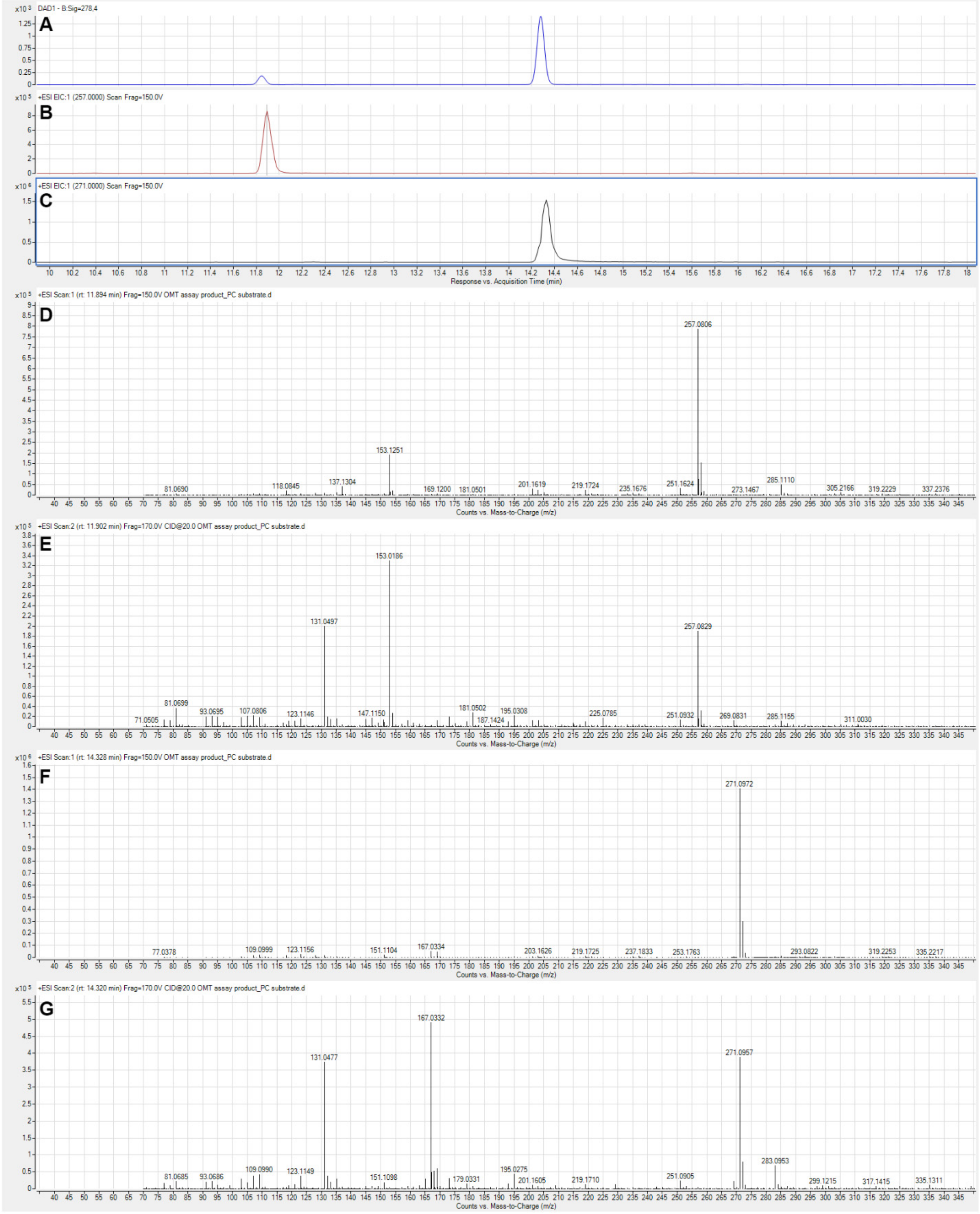

Figure S2. Representative ESI-LCMS data for EnOMT1 assay product with pinocembrin as substrate. (A) UV data; (B) Extracted Ion Current at *m/z* 257 $[M + H]^+$ for pinocembrin substrate; (C) EIC at *m/z* 271 $[M + H]^+$ for mono-methylated assay product; (D) MS1 data for pinocembrin substrate; (E) MS2 data for pinocembrin substrate showing key fragment ions (F) MS1 data for

monomethylated product; (G) MS2 data for monomethylated product showing key fragment ions.

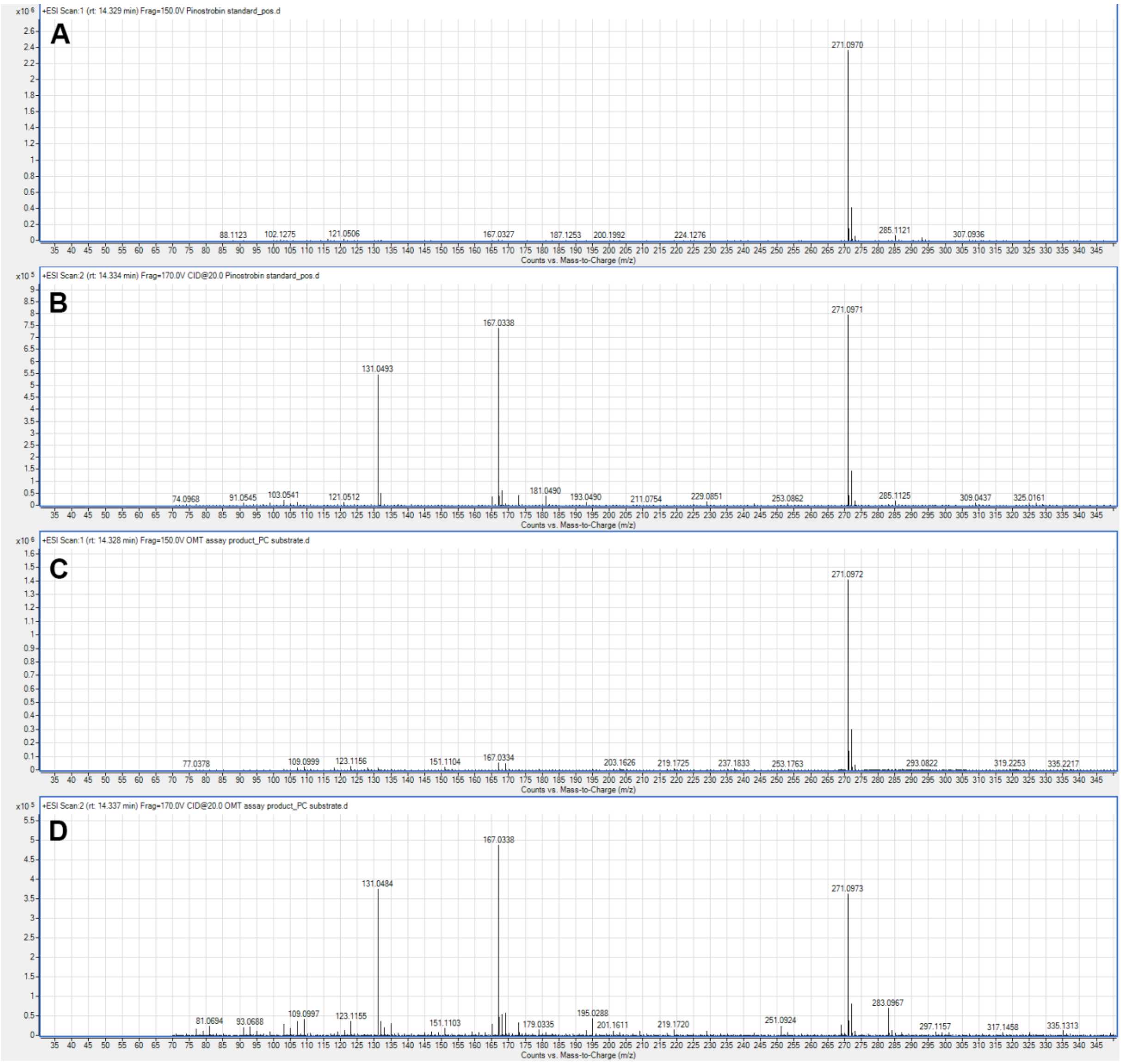

Figure S3. Representative ESI-LCMS data for EnOMT1 assay product with pinocembrin as substrate compared to a pinostrobin standard. (A) Extracted Ion Current at *m/z* 271 $[M + H]^+$ for the pinostrobin standard; (B) MS2 data for pinostrobin standard showing key fragment ions (C) EIC at *m/z* 271 $[M + H]^+$ for mono-methylated assay product; (D) MS2 data for monomethylated product showing key fragment ions matching those of the pinostrobin standard.

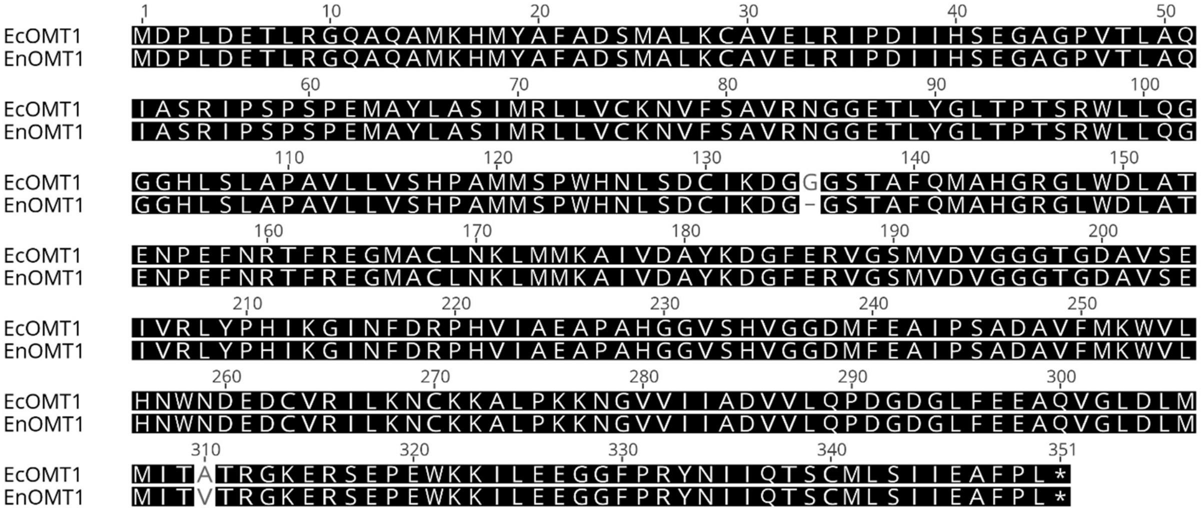

Figure S4. Amino acid alignment of OMT1 sequences from the pinostrobin-rich species *E. croajingolensis* (EcOMT1) and *E. nitida* (EnOMT1).

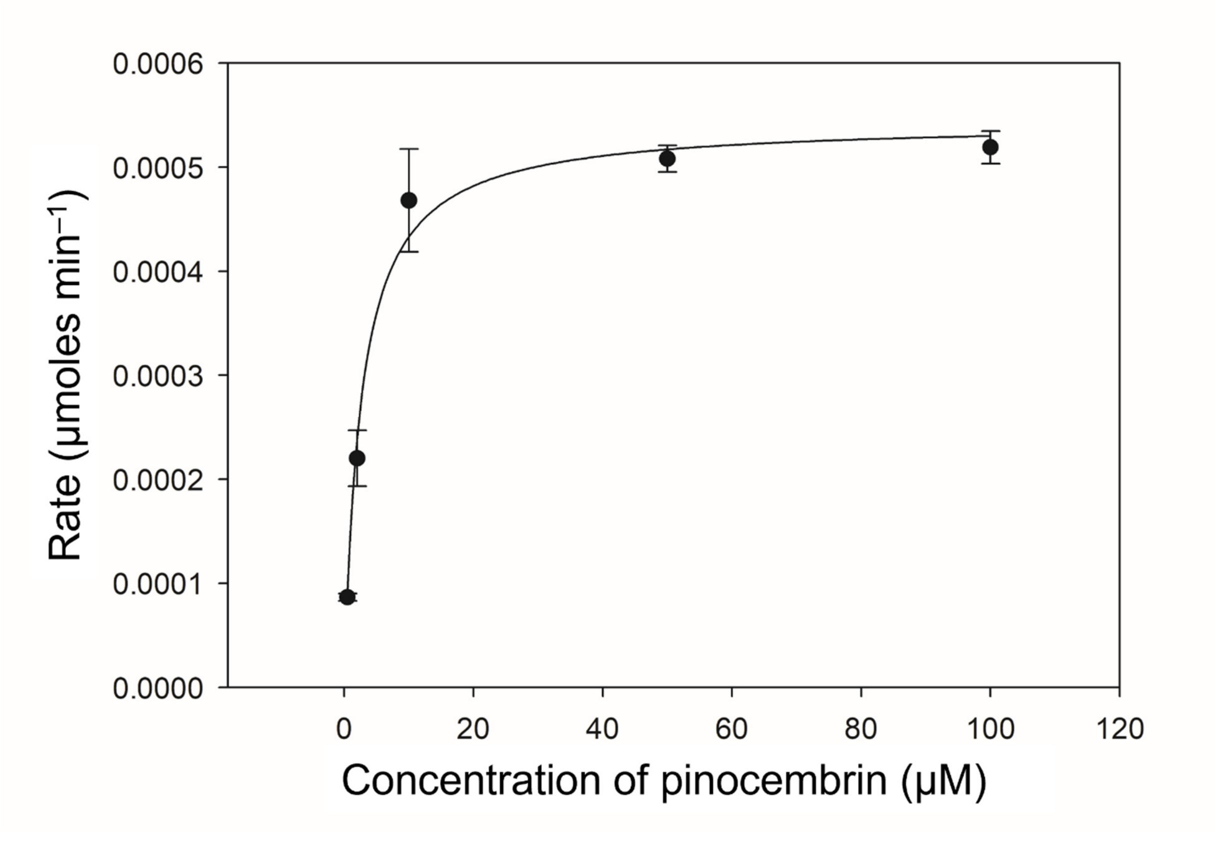

Figure S5. Michaelis-Menten curve of EcOMT1 with pinocembrin as substrate. Data points represent the initial velocities observed at different concentrations of pinocembrin at saturating SAM concentrations.

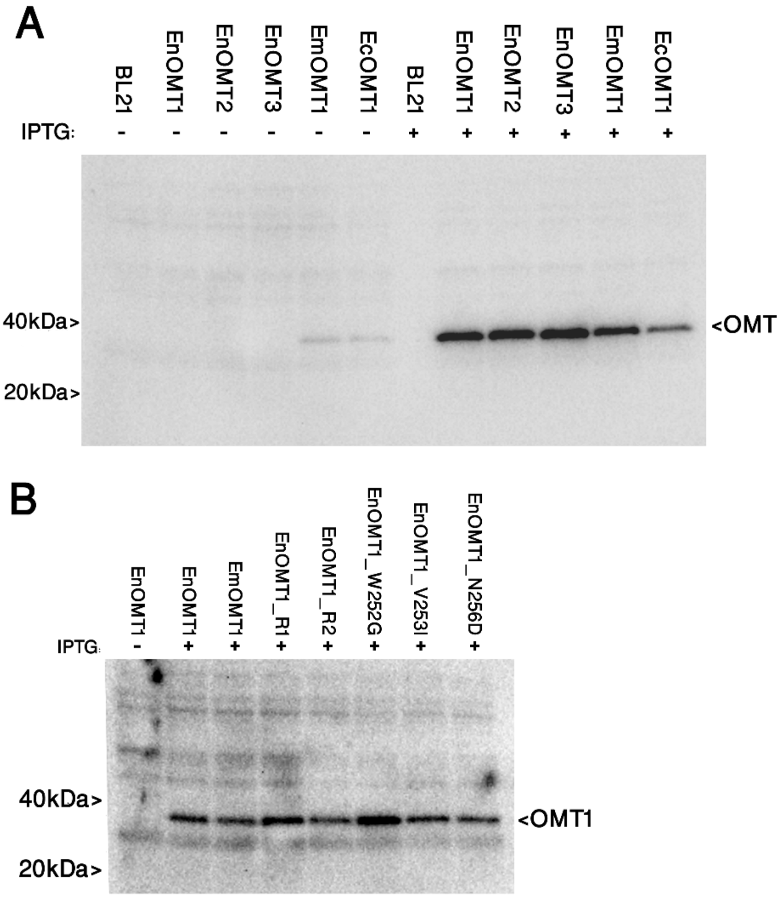

Figure S6. Western blot analysis of crude bacterial extracts containing recombinantly expressed OMTs. (A) Uninduced (IPTG -) and induced (IPTG +) samples of putative OMTs isolated from *E. nitida* (EnOMT1 -EnOMT3), *E. mitchelliana* (EmOMT1) and *E. croajingolensis* (EcOMT1), plus untransformed BL21 cells. (B) Uninduced (IPTG -) EnOMT1 and induced (IPTG +) EnOMT1, EmOMT1 and various EnOMT1 mutants. Bands marked as OMT(1) represent His-tagged *O*-methyltransferases (expected size 37-38kDa) detected by anti-His antibody.
