# Supplementary material for: Molecular Characterisation of Flavanone O-methylation in Eucalyptus
Source: Int J Mol Sci. 2022 Mar 16;23(6):3190. doi: 10.3390/ijms23063190 (PMC8954846; doi:10.3390/ijms23063190)
Supplement: Supplementary file 1 [file ijms-23-03190-s001.zip › Table S1_250222.pdf]

Table S1. List and details of primers used in the study.

Function	Primer name	Primer sequence	Notes
Cloning of EnOMT sequences into pHUE	EnOMT1_F	GCGGTACCATGGATCCTCTCGATGAGAC	KpnI restriction sequence underlined
	EnOMT1_R	TAGCGGCCGCTCACAGCGGGAAAGCTTCAA	NotI restriction sequence underlined
	EnOMT2_F	AGCCGGTACCATGAGCGAGAGCGAGATCTTC	KpnI restriction sequence underlined
	EnOMT2_R	ACCTGCGGCCGCTCAAGGATAACCTCAATG	NotI restriction sequence underlined
	EnOMT3_F	TTGAGAATTCATGGATCCTAGCGAGACCC	EcoRI restriction sequence underlined
	EnOMT3_R	AGCGAAGCTTTCACTTCTTTTTGTAAACTC	HindIII restriction sequence underlined
EnOMT1 mutagenesis	EnOMT1_R1_F	TCATGAAGGGGATCCTGCACGATTGGAAT	Nucleotide changes shown in red
	EnOMT1_R1_R	ATTCCAATCGTGCAGGATCCCCTTCATGA	Nucleotide changes shown in red
	EnOMT1_R2_F	GATGATCACGCTGAGCGGAGGTAAAGAG	Nucleotide changes shown in red
	EnOMT1_R2_R	CTCTTTACCTCCGCTCAGCGTGATCATC	Nucleotide changes shown in red
	EnOMT1_W252G_F	GTTTTCATGAAGGGGGTCCTGCAC	Nucleotide change shown in red
	EnOMT1_W252G_R	GTGCAGGACCCCCTTCATGAAAAC	Nucleotide change shown in red
	EnOMT1_V253I_F	TTTCATGAAGTGGATCCTGCACAATTGG	Nucleotide change shown in red
	EnOMT1_V253I_R	CCAATTGTGCAGGATCCACTTCATGAAA	Nucleotide change shown in red
	EnOMT1_N256D_F	GGTCCTGCACGATTGGAATGATG	Nucleotide change shown in red
	EnOMT1_N256D_R	CATCATTCCAATCGTGCAGGACC	Nucleotide change shown in red

qPCR analysis	Euccons4_F	TACAAGCGCTGTTGATATGTGGGC	qPCR reference gene
	Euccons4_R	TTGCCAATGAGGCGGATTCACAAG	qPCR reference gene
	EnOMT1_qPCR_F	TACAAGCGCTGTTGATATGTGGC	qPCR analysis of *EnOMT1* and *EmOMT1*
	EnOMT1_qPCR_R	TTGCCAATGAGGCGGATTCACAAG	qPCR analysis of *EnOMT1* and *EmOMT1*
